# Effect of Single Injection of Recombinant Human Bone Morphogenetic Protein-2-Loaded Artificial Collagen-Like Peptide in a Mouse Segmental Bone Transport Model

**DOI:** 10.1155/2019/1014594

**Published:** 2019-12-23

**Authors:** Ryo Tazawa, Hiroaki Minehara, Terumasa Matsuura, Tadashi Kawamura, Kentaro Uchida, Gen Inoue, Wataru Saito, Masashi Takaso

**Affiliations:** Department of Orthopaedic Surgery, Kitasato University School of Medicine, 1-15-1 Minami-ku, Kitasato, Sagamihara City, Kanagawa 252-0374, Japan

## Abstract

This study aimed to investigate whether a single injection of recombinant human bone morphogenetic protein-2-loaded artificial collagen-like peptide gel (rhBMP-2/ACG) accelerates consolidation at the bone defect site and bone union at the docking site in a mouse segmental bone transport (SBT) model. A critical sized bone defect (2 mm) was created in the femur of mice and subsequently reconstructed using SBT with an external fixator. Mice were divided into four treatment groups: Group CONT (immobile control), Group 0.2 (bone segments moved 0.2 mm/day for 10 days), Group 1.0 (bone segments moved 1.0 mm/day for 2 days), and Group 1.0/BMP-2 (rhBMP-2/ACG injected into the bone defect and segments moved 1.0 mm/day for 2 days). Consolidation at the bone defect site and bone union at the docking site was evaluated radiologically and histologically across eight weeks. Bone volume and bone mineral content were significantly higher in Group 0.2 than in Group 1.0. Group 0.2 showed evidence of rebuilding of the medullary canal eight weeks after surgery at the bone defect site. However, in Group 1.0, maturation of regenerative bone at the bone defect site was poor, with the central area between the proximal and distal bone composed mainly of masses of fibrous and adipose tissue. Group 1.0/BMP-2 had higher bone volume and bone mineral content compared to Group 1.0, and all mice achieved bone union at the bone defect and docking sites. Single injection of rhBMP-2/ACG combined with SBT may be effective for enhancing bone healing in large bone defects.

## 1. Introduction

Surgical treatment of large bone defects has long been a challenge for orthopaedic surgeons. Segmental bone transport (SBT) using an external fixator is a standard treatment for large-diameter bone defects at the donor site with low morbidity [1]. However, the long-term application of the device is needed for bone healing. In addition, 27% of patients who receive SBT treatment fail to show bone repair and union at the docking site and require additional procedures such as a second transport operation, autogenous bone grafting, and osteosynthesis [2]. Therefore, strategies that reduce the treatment time and improve new bone formation using SBT without the need for secondary surgery are necessary for the treatment of large bone defects.

Successful SBT treatment depends on both bone regeneration at the bone defect site, called the “regenerate,” and bone union at the meeting point between the transported and distal segments at the completion of bone transport, called the “docking site.” Studies aimed at improving the bone healing process in SBT have revealed that regeneration at the defect site is improved by the optimization of transport speed [3, 4]. However, nonunion at the docking site often occurs [5–7], even when consolidation is successfully achieved using an optimized transport speed. Therefore, additional optimization of SBT is required to repair large bone defects.

Bone morphogenetic protein-2 (BMP-2) is a potent inducer of bone formation [8]. Previous studies have reported the use of recombinant human (rh) BMP-2 in the treatment of open fractures and spinal surgery [9–11]. In a rabbit SBT model, BMP-2-loaded composite materials consisting of *β*-tricalcium phosphate (*β*-TCP) and polyethylene glycol (PEG) promoted consolidation and union at the docking site when injected percutaneously into the defect and docking sites following surgery and the completion of bone transport [12]. In clinical settings, however, repeated injection may increase the rate of such complications such as ectopic bone formation, seroma formation, and wound dehiscence [13, 14].

To date, several carriers to facilitate sustained release of growth factors have been developed [15, 16]. We previously reported that the artificial collagen-like peptide poly(POG)n was a useful carrier for growth factors. Injection of bFGF-loaded poly(POG)n into fracture sites stimulated periosteal bone formation in mouse fracture models [17, 18]. In the present study, we investigated whether a single injection of rhBMP-2-loaded artificial collagen-like peptide gel (rhBMP-2/ACG) accelerates consolidation at the bone defect site and bone union at the docking site in a mouse SBT model.

## 2. Materials and Methods

### 2.1. Implant*s*

**Figure 1:**
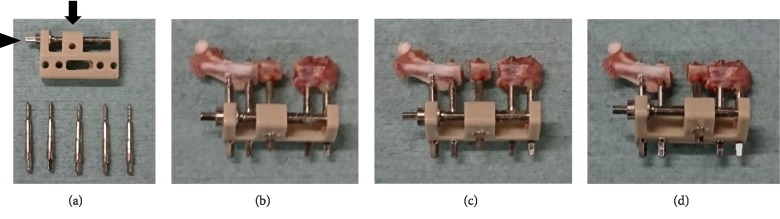
Segmental MouseDis. (a) The fixator block has a transport unit (black arrow) which can be moved distally along the transport track using an activation screw (arrow head). (b) Before bone transport. (c) Turning the activation screw clockwise causes the transport unit to move 0.2 mm along the transport track. (d) Completed bone transport.

Segmental MouseDis (Figure 1(a)), an external fixator device consisting of a fixator block and five 0.45 mm diameter mounting pins, was purchased from RISystem AG (Davos, Switzerland). The bone segment to be transported was attached to the device's transport unit via a mounting pin for movement across the defect (Figures 1(b)–1(d)).

### 2.2. Chemicals

CHO cell-derived rhBMP-2 was obtained from PeproTech Inc. (Rocky Hill, NJ, USA). Collagen materials have been used for the sustained release of rhBMP-2 in clinical settings [9–11]. We previously reported that collagen-like polypeptide poly(POG)n is superior to animal-derived collagen with regard to thermal stability and lack of pathogenicity and is a useful carrier which retains bFGF well [17, 18]. Our preliminary study showed that 2.0 *μ*g of rhBMP-2 without poly(POG)n failed to promote bone formation in a mice bone defect model. Therefore, to administer and retain rhBMP-2 at the bone defect site, we dissolved 2.0 *μ*g of rhBMP-2 in 22.5 *μ*L of the collagen-like polypeptide poly(POG)n gel, which was purchased from JNC Corporation (Tokyo, Japan).

### 2.3. Animals

All surgeries and handling were performed based on the guidelines of the Animal Ethics Committee of Kitasato University (Permission number: 2018-087). A total of 32 six-month-old male C57BL/6J mice (Charles River Laboratories Japan, Inc., Yokohama, Japan) were used for this study. The mice were fed a standard laboratory diet, CRF-1 (Oriental Yeast, Tokyo, Japan), and housed under controlled temperature (23 ± 2°C) and humidity (55 ± 10%) conditions and a 12-hour light/dark cycle. Mice were randomly divided into four treatment groups of eight mice each, namely, Group CONT (immobile control), Group 0.2, Group 1.0, and Group 1.0/BMP-2. A 2.0-mm critical sized bone defect was created in the right femur according to previous studies (see below for details) [19, 20]. In Group CONT, the defect was fixed with the Segmental MouseDis without transportation of the bone segment. Mice in Groups 0.2 and 1.0 underwent fixation with the device and the bone segment was moved 0.2 mm/day for 10 days and 1.0 mm/day for 2 days, respectively. Mice in Group 1.0/BMP-2 underwent fixation with the device and received an injection of 2.0 *μ*g of rhBMP-2 into the bone defect site immediately after the defect-creating surgery and the bone segment was moved 1.0 mm/day for 2 days.

### 2.4. Surgical Procedure

All mice were initially sedated using isoflurane followed by an intramuscular injection of domitor (Nippon Zenyaku Kogyo Co., Ltd., Fukushima, Japan), midazolam (Sand Co., Yamagata, Japan), and Vetorphale (Meiji Seika Kaisha, Ltd., Tokyo, Japan; 0.075 mL/100 g) at a ratio of 3 : 1 : 1. The operation was performed only on the right femur. The surgical site was prepared by hair removal and sterilization, and the surgery was performed under aseptic conditions. The skin of the lateral thigh was cut from the hip to the knee via a longitudinal incision 20 mm in length to expose the fascia latae between the gluteus superficialis and biceps femoris muscles. To implant the external fixator device, care was taken to position it at the center and parallel to the longitudinal axis of the femur. After predrilling, the first mounting pin was fixed to the distal segment of the femur by inserting it through the most distal hole in the fixator block. The second mounting pin was fixed to the proximal segment of the femur through the most proximal hole, holding the fixator block parallel to the femur. The remaining three mounting pins were inserted through the final three holes, including one in the transport unit.

After adjusting the fixator block, a transport segment was created by performing a transverse osteotomy between the second and third pins from the proximal end using a 0.22 mm diameter Gigli saw. A 2.0 mm bone defect was then created between the third and fourth pins from the proximal end using a microdrill. Only mice in Group 1.0/BMP-2 received an injection of 2.0 *μ*g of rhBMP-2 into the 2 mm bone defect site. Finally, nonabsorbable thread was used to suture the fascia and skin. Successful completion of the surgical procedure was validated by examining radiographs. All mice were free to perform normal activities immediately after surgery. In all groups except Group CONT, bone transport was conducted routinely from two days after surgery. All animals were sacrificed at eight weeks after surgery. The femur with external fixator was carefully dissected out for radiological and histological evaluation.

### 2.5. Soft X-ray Radiographs

The process of SBT and regenerative new bone formation were monitored using soft X-ray radiographs (SOFTEX-CMB4; SOFTEX Corporation, Kanagawa, Japan) taken at an exposure of 10 seconds, a voltage of 35 kV, and a current of 3.0 mA using X-ray IX Industrial Film (Fuji Photo Film Co., Ltd., Tokyo, Japan).

### 2.6. Microcomputed Tomography (Micro-CT)

Following the sacrifice of mice eight weeks postsurgery, femurs were extracted and fixed in 4% paraformaldehyde for 48 hours at 4°C. The tissue was subsequently moved to PBS and imaged on a microfocus X-ray CT system (inspeXio SMX-90CT; Shimadzu, Tokyo, Japan). Tube voltage, tube current, and voxel size were 90 kV, 100 *μ*A, and 30 × 30 × 30 *μ*m, respectively. 3D imaging software (TRI/3D BON; Ratoc System Engineering Co., Ltd., Tokyo, Japan) was used to generate 3D reconstructed images at a threshold determined based on discriminant analysis. We evaluated bone union at both the bone defect and docking sites. Bone union was defined as the continuity of cortical bone over three of four images in the sagittal and coronal plane at the center of the bone defect site and docking site, respectively. We also calculated the bone volume (BV) and bone mineral content (BMC) of regenerative new bone at the bone defect site in all samples. All parameters were measured in a rectangular region of interest (ROI) that consisted of a 1500 *μ*m length of bone mass indicative of regenerative new bone between the proximal femoral segment and transport segment.

### 2.7. Histology

Following micro-CT imaging, femurs were submerged in 20% EDTA for four weeks for demineralization. The resulting tissue was embedded in paraffin using an automatic tissue processor (Tissue-Tek VIP 6; Sakura Fine Tek, Tokyo, Japan) and sectioned through the femur's long axis at 3 *μ*m in the sagittal plane using a microtome (REM-710; Yamato Kohki industrial Co. Ltd., Saitama, Japan). All sections were then stained with hematoxylin and eosin (HE) and evaluated qualitatively.

### 2.8. Statistical Analysis

SPSS software (version 19.0; SPSS, Chicago, IL, USA) was used for all analyses. Differences between groups were analyzed using one-way ANOVA and a subsequent Bonferroni's post hoc comparisons test. *p* < 0.05 was considered significant.

## 3. Results

### 3.1. Radiological Evaluation

#### 3.1.1. Soft X-ray Radiographs

**Figure 2:**
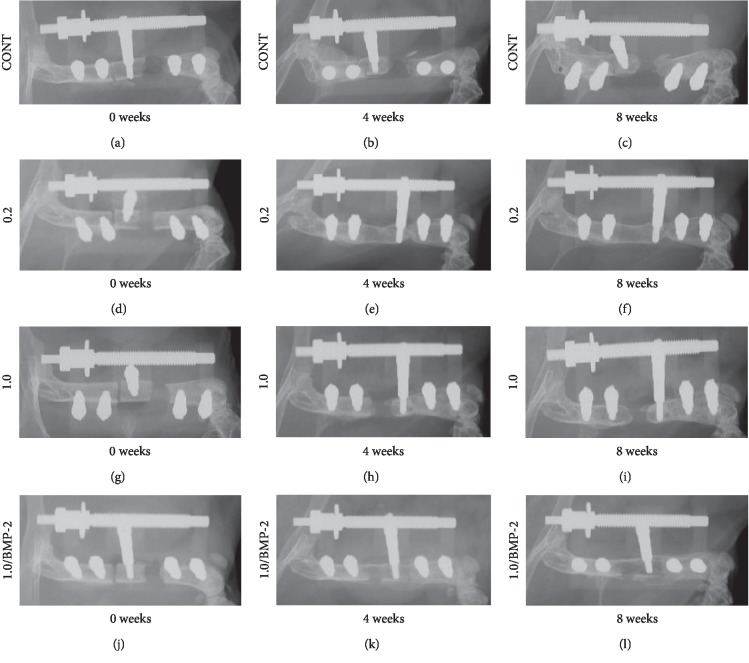
Soft X-ray images of the process of SBT and regenerative new bone formation. (a–c) Group CONT. (d–f) Group 0.2. (g–i) Group 1.0. (j–l) Group 1.0/BMP-2. Radiographs were obtained immediately after surgery (a, d, g, j) and 4 (b, e, h, k) and 8 (c, f, i, l) weeks following surgery.

To evaluate the process of SBT and regenerative new bone formation, we examined soft X-ray radiographs (Figure 2). At four weeks after surgery, Group 0.2 and Group 1.0/BMP-2 showed a weak shadow indicating regenerative new bone at the bone defect site that gradually consolidated over time. At eight weeks after surgery, Group 0.2 and Group 1.0/BMP-2 showed definitive evidence of regenerative new bone formation. In contrast, Group CONT and Group 1.0 showed nonunion at the bone defect site.

#### 3.2.2. Micro-CT

**Figure 3:**
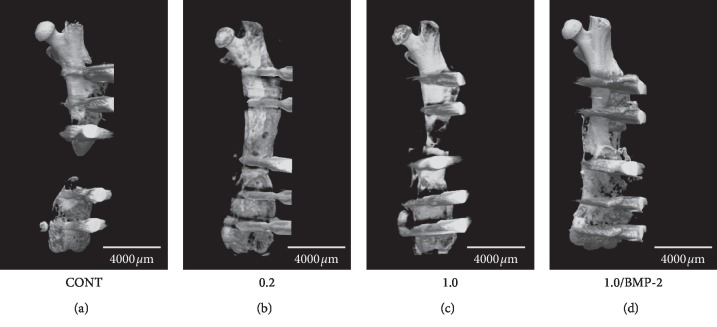
Microcomputed tomography images of untreated and treated femurs in mice with a 2 mm bone defect at 8 weeks after surgery. (a) Group CONT. (b) Group 0.2. (c) Group 1.0. (d) Group 1.0/BMP-2. The scale bar indicates 4000 *μ*m.

**Figure 4:**
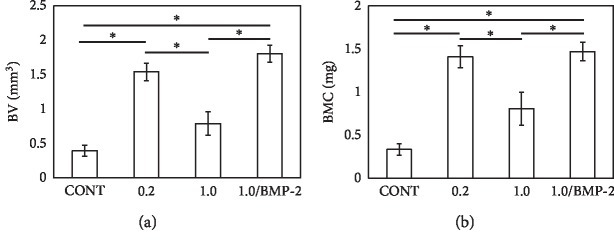
Analysis of new bone formation in mouse femurs at 8 weeks after surgery from microcomputed tomography images. (a) Bone volume (BV) and (b) bone mineral content (BMC) at the bone defect site. Data show mean ± standard error (S.E.). *n* = 8. ^*∗*^*p* < 0.05.

To evaluate bone union at the bone defect and docking sites, we performed micro-CT (Figure 3 and Table 1) and calculated the BV and BMC (Figure 4) at eight weeks after surgery. At the bone defect site, BV and BMC were significantly higher in Group 0.2 than in Group CONT (BV, *p* < 0.001; BMC, *p* < 0.001) and seven of eight mice showed bone union. In contrast, BV and BMC were not significantly different in Group 1.0 compared to Group CONT (BV, *p*=0.246; BMC, *p*=0.098) and two of eight mice showed bone union. At the docking site, four of eight mice in Group 0.2 and five of eight mice in Group 1.0 showed bone union. On the other hand, Group 1.0/BMP-2 showed significantly higher BV and BMC than Group CONT and Group 1.0 (BV, *p* < 0.001 and *p* < 0.001; BMC, *p* < 0.001 and *p*=0.007, respectively). In addition, all mice in Group 1.0/BMP-2 showed bone union at the bone defect and docking sites.

### 3.2. Histological Evaluation

**Figure 5:**
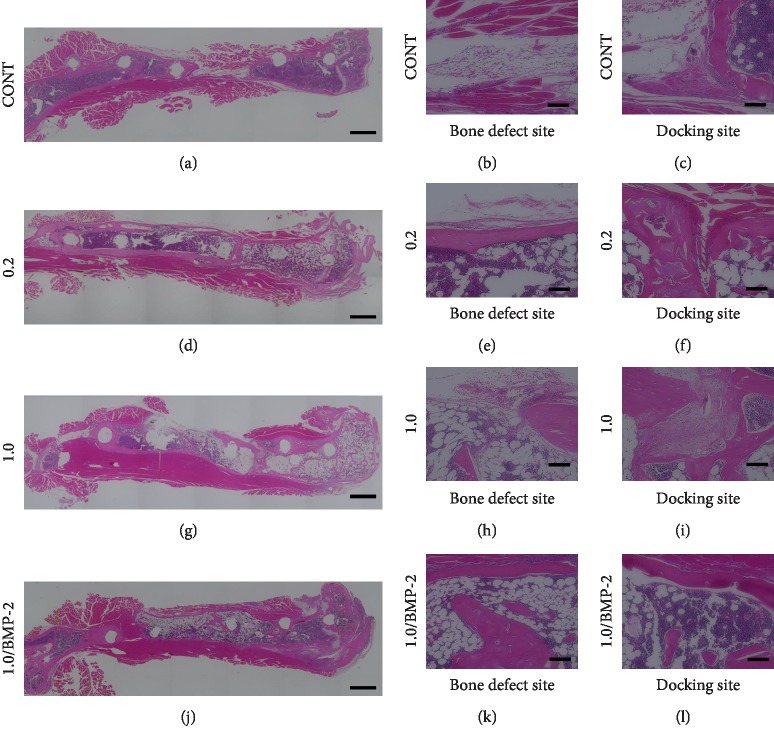
Hematoxylin and eosin-stained tissue sections of femurs showing new bone formation at the bone defect site and union at the docking site at 8 weeks after surgery. (a–c) Group CONT. (d–f) Group 0.2. (g–i) Group 1.0. (j–l) Group 1.0/BMP-2. The scale bars indicate 1000 *μ*m (a, d, g, j) and 100 *μ*m (b, c, e, f, h, i, k, l).

We performed a histological examination to evaluate new bone formation (Figure 5). HE staining of tissue from Group 0.2 and Group 1.0/BMP-2 showed large amounts of longitudinal trabecular bone and regenerative new bone and evidence of rebuilding of the medullary canal at eight weeks after surgery at the bone defect site. Meanwhile, in Group CONT and Group 1.0, maturation of regenerative bone at the bone defect site was poor, with the central area between the proximal and distal bone composed mainly of masses of fibrous and adipose tissue. At the docking site, all mice in Group 1.0/BMP-2 showed bone union between the distal end of the transported segment and distal bone. In contrast, Group 0.2 and Group 1.0 showed discontinuity between the transported segment and distal bone with fibrocartilaginous tissue.

## 4. Discussion

We used a mouse bone transport model to study the effects of a single injection of rhBMP-2/ACG on bone regeneration at the bone defect site and bone union at the docking site. All mice treated with a single injection of rhBMP-2/ACG exhibited accelerated bone healing at both the defect and docking sites, even with rapid bone transport.

Previous studies have shown that the optimization of transportation speed in SBT improves the consolidation of regenerative new bone formation. For example, Ilizarov [3, 4] demonstrated that the optimal distraction rate was 1.0 mm/day, while a rate of 0.5 mm/day resulted in premature bone healing and a rate of 2.0 mm/day produced only fibrous connective tissue at the distal ends of the bone without osteogenesis in a canine tibia SBT model. In contrast, increased time to docking often results in nonunion at the docking site with interposed fibrocartilaginous tissue at the bone ends [5]. Consistent with these reports in human and large animal studies, our mouse model showed that consolidation was achieved using a distraction rate of 0.2 mm/day, while consolidation was insufficient using a rate of 1.0 mm/day. Union at the docking site was inadequate at the faster distraction rate, and fibrocartilaginous tissue was observed histologically. Our results suggest that findings from mouse SBT models may be extrapolated to larger animal models.

rhBMP-2 administration is safe and increases the rate of healing of fractures and wounds and reduces infection rates in patients with open tibia fractures [9]. A previous study reported the effectiveness of a BMP-2-loaded *β*-TCP/PEG composite injected into the distraction gap at the start and into the docking site at the end of distraction in rabbit models [12]. In the present study, we demonstrated that a single injection of rhBMP-2/ACG combined with SBT accelerated bone healing at both the defect and docking sites in a mouse critical sized bone defect model, even at a faster distraction rate. rhBMP-2/ACG combined with SBT may therefore be effective for enhancing bone healing in large bone defects and reduce the treatment period. However, nonclinical research in nonhuman primates followed by clinical trials is needed to optimize the rhBMP-2 dose required to facilitate distraction osteogenesis in patients with diaphyseal bone defects.

## 5. Conclusions

A single injection of rhBMP-2/ACG exhibited accelerated bone healing at both the defect and docking sites, even with rapid bone transport, in a mouse SBT model. The use of rhBMP-2/ACG combined with SBT may accelerate bone healing in large bone defects without the need for repeated BMP-2 injection.

## Figures and Tables

**Table 1:** Number of mice in each group showing consolidation at the 2 mm bone defect and union at the docking site.

	Consolidation rate at the bone defect site	Union rate at the docking site
CONT	0/8	—
0.2	7/8	4/8
1.0	2/8	5/8
1.0/BMP-2	8/8	8/8

## Data Availability

The datasets supporting the conclusions of this article are included within the article. The raw data can be requested from the corresponding author.

## Supplementary Materials

Supplementary Materials The ARRIVE checklists were filled completely to improve the reporting of research using animals.
